# Supplementary effect and durability of prototype insecticide-treated eave curtains on indoor resting mosquitoes in Kadibo division, Western Kenya

**DOI:** 10.5281/zenodo.10818166

**Published:** 2016-08-16

**Authors:** Martin T.O. Odhiambo, John M. Vulule, Yaw A. Afrane, Maurice Ombok, Rune Bosselmann, Ole Skovmand

**Affiliations:** 1 Centre for Global Health Research, Kenya Medical Research Institute, PO Box 1578, Kisumu, Kenya; 2 Jaramogi Oginga Odinga University of Science and Technology, PO Box 210-40601, Bondo, Kenya; 3 Intelligent Insect Control (IIC), 118 Chemin des Alouettes, Castelnau-le-Lez, France

## Abstract

**Background:**

Use of insecticide treated bednets (ITNs) was a breakthrough in the fight against malaria. However, ITNs are only effective when properly used. Recent reports indicate low compliance in ITN usage and changes in biting times of malaria vectors with early and late biting cases recorded when people are not sleeping under their nets. Hence, there is a need to develop methods to supplement or replace the use of ITNs for malaria vector control. A field trial was conducted to investigate the effect and longevity of prototypes of long lasting impregnated UV protected eave nets, curtains and door hangers (fully screened houses), compared to houses with bednets only, in traditional East African houses.

**Materials and methods:**

A randomised controlled trial was carried out in the Ahero rice irrigation scheme in Nyando district, Western Kenya. Eighty houses with open eaves were randomly selected. Forty of these houses were fully screened (FSH+LLINs) with long lasting insecticide-treated nets/curtains used to screen the eaves, windows and doors. The FSH materials were produced with anti-UV additives. The other 40 houses served as controls. Long lasting insecticide-treated bednets (LLINs) were suspended over all sleeping areas in the control and intervention houses. Indoor resting *Anopheles* mosquitoes were collected using pyrethrum spray catches (PSC) during both dry and wet seasons. Indoor population densities of anophelines were compared between intervention (FSH+LLINs) and control (LLINs) houses. Loss of insecticide (deltamethrin) was compared after 12 and 24 months for both the FSH materials and LLINs through bioassays and chemical analyses.

**Results:**

In the FSH+LLINs houses densities of indoor resting *Anopheles funestus* and *An. arabiensis* were reduced by 82% (RR=0.18, 95% CI 0.09-0.36, P<0.0001) and 70% (RR=0.30, 95% CI 0.15-0.58, P<0.0001), respectively. No significant difference was recorded for indoor resting *Culex* spp. (RR=0.95, 95% CI 0.48-1.86, P=0.8). The population of indoor resting bloodfed *An. arabiensis* and *An. funestus* was reduced by 72% (RR=0.22, 95% CI 0.09-0.51, P<0.0001) and 84% (RR=0.16, 95% CI 0.07-0.33, P<0.0001) in the FSH+LLINs houses and LLIN houses, respectively. Insecticide loss in eave nets did not depend on the side of the house where the nets were placed. The eave nets showed little loss of bio efficacy over the 12-24 months period.

**Conclusions:**

The study revealed that the use of insecticide-treated nets on the eaves and windows combined with door hangers largely impeded entrance of anopheline mosquitoes into houses and can be used to compliment LLINs for household protection. The eave nets were suspended from wood structures near the eave and remained in place when walls were re-plastered. The nets are therefore not depending on daily compliance behaviour and provide protection for the entire household.

## 1 Introduction

Vector control remains the most effective measure to control malaria transmission and is therefore one of the core strategies for global malaria control [1]. There are many efforts focused on preventing man-vector contact and the widely embraced options are large-scale implementation of long lasting impregnated nets (LLINs) and indoor residual spraying (IRS) [2]. An LLIN with a person(s) sleeping inside acts as a trap that will prevent biting and kill the blood-questing malaria vectors if they come into contact with the net for sufficient time. This will prevent the vector from biting humans and transmit *Plasmodium* parasites. However, bednets can only protect people if used correctly during the night and mosquitoes may also bite earlier in the evening [3-5]. Studies have shown that the primary motivation for people to use nets and accept IRS is protection against mosquito nuisance more so than for disease prevention [6-8]. While appropriate and consistent use is essential in preventing malaria, LLIN use often lags behind LLIN ownership, especially when and where mosquito nuisance is low [9]. Bednets also quickly get physically damaged and studies have shown that physical damage is more important for net discarding and failure than loss of insecticide [9,10]. Resurgence in malaria incidence has been recorded in several health facilities in Western Kenya despite the free distribution of and advocacy on the use of long lasting insecticide nets [11]. This has been attributed to behavioural changes in biting time of the malaria vectors and also to the lack of compliance in bednet use when mosquito nuisance declines [6].

House design has been established as a significant factor affecting mosquito entry into houses [12]. Screening of house openings, such as doors, windows and eave spaces can reduce mosquito densities and malaria cases in these households [13-16]. Hence, screening against mosquito entry has long been recommended as a way of reducing human exposure to mosquito bites and infection with malaria. In many rural parts of Africa, houses are simple structures with a gap (open eave) between the walls and the roof which provides illumination and ventilation. This gap also allows *Anopheles* mosquitoes to enter attracted by carbon dioxide and human odour [11]. Blocking this gap with a semi-permeable barrier like mosquito netting still allows air and light to enter the house but prevents mosquito entry, which been shown to be effective in reducing malaria transmission in Burkina Faso [15-17]. However, studies where bednet material was used and set up quickly to cover the eave spaces showed little protection in contrast to when a more thorough, slower and therefore more expensive implementation of such nets was undertaken [16]. Therefore, nets should be installed fast and properly for them to offer protection at a fair cost.

Materials for screening a house are exposed to ultraviolet (UV) from the sunlight as opposed to bednets, which are used indoors. Due to the destructive properties of UV-light to insecticides used in the treatment of bed-nets, manufacturers advise users to dry nets in the shade after treatment or washing (Bestnet for the Netprotect® used in this study). In a trial in The Gambia, to prevent breakdown of the permethrin on the nets by UV light, the nets were dried in the shade after impregnation [18]. Studies carried out in Iran on bednets exposed to sunlight for 3 days between washes showed a significantly greater loss on insecticide deposits when compared with control nets [19]. It may therefore be advantageous to use UV protection additives to materials for eave nets, curtains and door hangers.

This study focused on the feasibility of screening houses compared to using long lasting bednets only. The screening materials were followed for two years, where insecticide survival in the materials used for house screening and in bednets was analysed. Biological efficacy was also measured using WHO cone test on the materials. Entomological impact was tested by comparing the indoor resting densities of mosquitoes in a cohort of houses in the intervention area (where houses were screened) to control houses. The net and laminate material were designed and produced specifically for this use by Intelligent Insect Control (IIC), based on our experience from a small, preliminary study in the same area. Local villagers and technicians were trained in setting up the material properly in very short time.

## 2 Materials and methods

### 2.1 Study site

The study was carried out in Kobura village in Ahero (0° 1833"N 34°91'67"E), Kisumu county, Western Kenya. It is located 24 kilometres southeast of Kisumu town with a population of 8,788 people [20]. Average annual rainfall is 1082 mm with maximum precipitation between March and July and a less intense rainfall season between September and October. The driest period is between December and February. Main economic activities include rice cultivation using large-scale irrigation at the border of the villages, and cultivation of maize, sorghum and cassava. Other activities include fishing in Lake Victoria. Houses in the area are typically constructed with a wood framework with mud walls and grass thatched or corrugated iron roof. The eaves of most houses are open allowing for unimpeded entry and exit of mosquitoes. Most houses also have windows without glass. A family compound consists of the main house for the parents and one or more houses for adolescent and married male siblings. Malaria is highly endemic in the region and transmitted throughout the year. The principal mosquito vectors are *Anopheles gambiae s.s.*, *An. funestus* and *An. arabiensis* [12,21].

### 2.2 Sample size determination

Forty houses from the intervention village and 40 houses from the neighbouring control village were followed for 3 months before the intervention to determine mosquito density per house. The number of houses to be sampled per day from control and treatment houses for indoor resting mosquito collection was determined based on the entry rate per night in this pre-treatment period. An average of 20 mosquitoes entered each night, and we expected a 75 % reduction as screening effect. Based on this, 8 houses in each block were to be followed to provide significant data assuming normal distribution of data and a 5 % significance level.

### 2.3 House selection criteria

**Figure 1.**
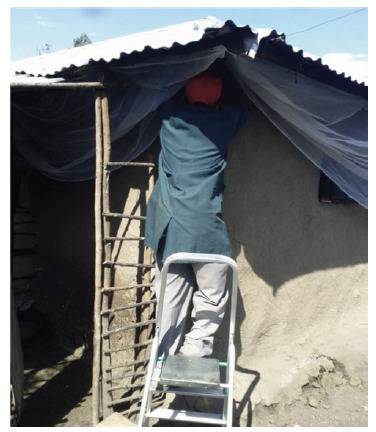
Installation of eave netting under the roof.

**Figure 2.**
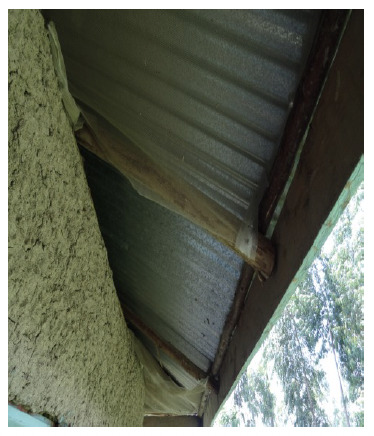
Model house with fitted eave netting.

**Figure 3.**
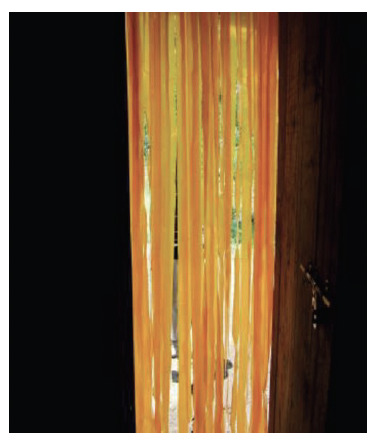
Insecticide-treated door lamellae.

**Figure 4.**
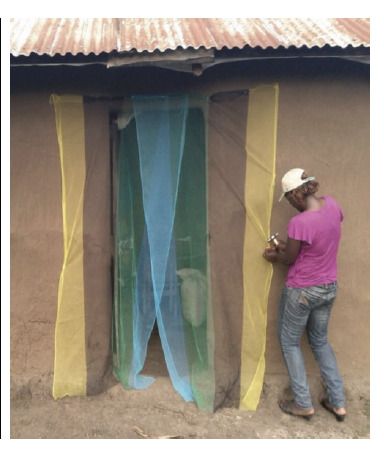
Overlapping pieces of LLIN material to prevent house entry by mosquitoes.

The study was a randomised experimental design incorporating separate intervention and control areas for mosquito sampling. A total of 40 houses from each arm, similar in size, with open eaves, corrugated iron sheet roofs, mud walls and 2 sleepers inside were selected using random selection in two adjacent areas (Sidho and Kobura villages) in Nyando District. The exact numbers of doors and windows in each of the selected houses were recorded. The houses were marked with a unique number on the on the doorframe for identification. Bednets (LLINs, Netprotect®, Bestnet Aps) were provided for all sleeping places for the selected houses. In the treatment arm, houses were fully screened (FSH) with polyethylene nettings fixed on the eaves (Figure 1 and 2) and nets or door hangers for windows and doors. The materials were formulated with deltamethrin with high level of UV protection (Netprotect®, Bestnet Aps). For window curtains, polyester and polyethylene (PE) were used alternatingly to observe preferences (to be published separately). These materials were provided by IIC. Window curtains were suspended by fixing the top side on the mud walls with nails and leaving the bottom side free, but extending well below the opening. This was done to allow people to open and close wood frames covering windows and thus assure that the curtains remained in place based on personal observations by one of the authors from earlier net curtain project in Burkina Faso, where nets were fixed to the walls and released by the inhabitants (though the authors of the paper never mentioned so). Two different models of door hangers were used: 0.5 mm thick laminate of low density polyethylene with incorporated deltamethrin (referred to as laminated door streamers), cut at the factory into 7 cm wide stripes except at the top (Figure 3), or polyethylene nets made from the same material as the eave nets, both delivered by IIC. When laminated door streamers were used, a double layer of two pieces of 100 cm width were fixed to cover the doorway in the way the lamels on one layer covered the cuts of the other. For the polyethylene nets, two overlapping pieces of net were hung from above the doorway (Figure 4). In the control houses only LLINs were suspended above all sleeping places.

Two designs of LLINs (Netprotect®) were provided to all selected houses in treatment (FSH+LLINs) and control (LLINs) areas. Normal, fix beds were given a rectangular net, whereas those sleeping places with mattress on the floor were offered a choice between a standard rectangular bed net and a triangular “A-shaped” net. The “A-shaped” net hangs from a string from 3 plastic rings and can be pushed to one end of the string to be out of the way during daytime. The A-net was designed for people, mostly small children, sleeping in areas that are used for other activities during daytime (e.g. living rooms). In these rooms the mattress is rolled out on the floor in the evening and stored away during the day. These sleeping places have very low use of bednets even in areas where ITN use is otherwise high [18]. Acceptance data for the two forms of bednets will be published later.

By training personnel and using ladders and staple guns, the set up time of eave nets was reduced to 30 min when 2 people installed the nets of the eaves and a third person installed window curtains and door hangers.

### 2.4 Mosquito sampling

During the pre-trial survey of 3 weeks, indoor resting mosquitoes were collected using pyrethrum spray catch (PSC) [22] in selected houses twice a week to establish the baseline entry rate for the two areas. After intervention a total of 16 houses of similar size, with 2 sleepers inside were randomly selected from the 80 houses, by sampling 8 houses from each arm, every second week of the month for 6 months. The first mosquito collections were made during the dry months of December- February 2011, while the last 3 collections were done in the wet months from April to June 2012. Mosquito sampling was carried out between 7 and 10 am. Knocked-down mosquitoes collected from each house were put on moist filter paper in petri dishes. Collected mosquitoes were sorted by sex and species, using morphological characteristics according to Gillies and Coetzee [23] and Gillies and De Meillon [24] at KEMRI in Kisumu. Females were further sorted according to their respective blood feeding stages (unfed, bloodfed, semi-gravid or gravid) by examining their abdomen under a microscope. All bloodfed mosquitoes from each collection were preserved in labelled vials containing anhydrous calcium sulphate awaiting species identification, PCR and host bloodmeal identification.

### 2.5 Effect of sunlight on chemical attrition of the nets

To analyse the effect of UV light on the deltamethrin content in relation to the direction of the sun, pieces (25x25cm) of nets were cut from the eave-nets and door-nets from sides facing towards the east or north side of the houses and compared with LLIN bednet samples. Eave nets are positioned under the roof and therefore may only be temporarily exposed to mild sunlight at dawn (east side) and dusk (west side). Netting pieces were cut from FSH houses after 12 and 24 months and analysed for residual deltamethrin content. Each piece of material collected was wrapped individually in aluminium foil and labelled according to house identification number and the side of the house from which it was collected. The netting pieces were stored at 24°C in the laboratory before being subjected to extraction in xylene under reflux followed by direct injection in a gas chromatograph with ion detection (GC-FID), a method developed by the reference laboratory for WHO Gembloux [25,26], though the author used GC-ECD. Assays at the beginning of the trial (time 0) provided the baseline insecticide content in the nets. Two years later, another batch of netting pieces were cut from the eave nets, door curtains and bednets and the deltamethrin content determined. These analyses were conducted by the Intelligent Insect Control laboratory in Hanoi, Vietnam (now Vegro Laboratories).

### 2.6 Determination of residual biological activety of the nets

Sample pieces measuring 30x30 cm were cut from the eave nets after 12 and 24 months and from door hangers and bednets after 24 months. The samples were wrapped in aluminium foil, stored in a cool box and transported to KEMRI laboratory for bioassays. A 40x40 cm piece of netting was cut from a replacement roll of netting and stapled to cover the hole in the eave net. Bednets were replaced (only after 24 months). Efficacy of net samples was determined using a standard WHO cone held in place using a plastic manifold. For each net piece (sub-sample), five laboratory bred susceptible *Anopheles gambiae* (Kisumu strain) mosquitoes (sugar fed, 2-5 day old) were introduced into each cone and exposed for 3 minutes. The test was replicated for a total of 20 mosquitoes per net piece (40 mosquitoes per net). After the exposure, the mosquitoes were gently transferred from the cones using aspirators and kept in plastic cups on which cotton with a 10% glucose solution was placed. Knockdown was recorded after 1 hr, mortality after 24 hrs. Untreated nets were used as controls. The bioassays were carried out at 27±2 ° C and 80±10% relative humidity according to WHO protocol [25]. Data were recorded in a structured form for further statistical analysis.

### 2.7 Data analysis

All variables were evaluated using a Poisson regression model at the level of household (Proc-Genmod SAS 9.2). The dependent variable was the number of mosquitoes collected. Generalised estimating equation (GEE) with an exchangeable working correlation matrix was used to control clustering at the household level because the collection was done more than once in the same houses. The variable found to have P-value less than 0.1 in univariate analysis were included in the initial multivariate model. The final model only retained variables significant at the level of P<0.05. Male mosquitoes were not included in the final analysis. Paired sampled t-test (Proc t test SAS ver 9.2 for windows) was used for the comparison of residual deltamethrin content. Bio efficacy of the material over time was measured by calculating the percentage knock down and 24 hr mortality following mosquito exposures.

### 2.8 Ethical review

The study was approved by the Scientific Steering Committee and the Ethical Review Committee of KEMRI (approval number 2145) before commencement of the study. Local approval was granted by the County commissioner of Kisumu County. Written informed consent was obtained from heads of households after explaining the objectives of the study in the local language to them.

## 3 Results

### 3.1 Mosquito indoor resting density composition and abundance

During the pre-intervention survey, 526 mosquitoes were collected from the selected houses through biweekly PSC catches for 3 weeks. Out of these, 291 were collected from the houses that were going to receive the FSH+LLINs and 239 from the houses that were going to receive LLINs only. Of the 291 indoor resting mosquitoes from the later FSH+LLINs houses, *A n. gambiae s.l.* comprised 150 (51.6%)*, An. funestus* 92 (31.6%) and culicines 49 (16.8%)*.* In the control cluster, 137 (57.3%) were *An. gambiae s.l.,* 82 (34.3 %) were *An. funestus* and 20 (34.3 %) were Culicines. There was no significance difference in the mean densities of indoor resting *A n. gambiae s.l.* (P=0.45) and *An. funestus* (P=0.80) mosquitoes in the FSH+LLINs and LLINs houses.

### 3.2 Evaluation of the trial

During the trial period, a total of 926 indoor resting mosquitoes were collected. These comprised 755 (81.5%) anophelines and 171 (18.5%) *Culex* mosquitoes*.* Of the 755 anophelines, 134 (17.7%) were collected from FSH+LLIN houses compared to 621 (82.3%) in the LLIN-only houses. Molecular identification of the *A n. gambiae s.l.* by PCR showed that all specimens were *An. arabiensis*. Anophelines in the FSH+LLIN houses comprised 73 (55%) *An. arabiensis* and 61 (45%) *An. funestus*. In the LLIN-only houses, *A n. arabiensis* numbered 328 (53%) and *A n. funestus* 293 (47%). The population of indoor resting bloodfed anophelines comprised of 36 (27%) of the mosquitoes collected in the FSH+LLIN houses compared to 266 (42.8%) the LLIN-only houses. The bloodfed anophelines in the FSH+LLIN comprised of 14 (19%) *An. arabiensis* and 22 (36%) *An. funestus*. Whereas in the LLIN-only houses, 112 (25%) were *A n. arabiensis* and 154 (53%) *An. funestus*. The reduction in culicines was observed There was no significant difference in the population of indoor resting culicine mosquitoes in the FSH + LLIN houses 91 (53%) compared to the LLIN-only houses 80 (47%; P=0.87).

Univariate analysis (Table 1) showed that the overall density of indoor resting anopheline mosquitoes was reduced by 77% (RR=0.23, 95% CI 0.04-0.39, P<0.0001). Densities of indoor resting *An. funestus* and *An. arabiensis* in the FSH+LLIN houses were reduced by 82% [RR=0.18, 95% CI 0.09-0.36, P<0.0001] and by 70% [RR=0.30 95%, CI 0.15-0.58, P<0.0001] respectively. No significant difference was recorded for indoor resting *Culex* spp. [RR=0.95, 95% CI 0.48-1.86, P=0.8]. The population of indoor resting bloodfed *A n. arabiensis* and *A n. funestus* in the FSH+LLIN houses was significantly reduced by 72% [RR=0.22, 95% CI 0.09-0.51, P<0.0001] and 84% [RR=0.16, 95% CI 0.07-0.33, P<0.0001], respectively, compared to LLIN-only houses.

**Table 1.** Effects of the different treatments on indoor resting mosquitoes in Kadibo Division.

	Category	Species	Mean Density (95% CL)	Univariate (RR 95%)	Multivariate (RR 95%)
Treatment	FSH+LLINs	Anophelines	1.46 (0.80-2.11)	0.23(0.04-0.39)***	0.25(0.15-0.42)***
	LLIN-only		6.38 (4.49-8.26)	Ref	Ref
	FSH+LLINs	*An. arabiensis*	0.75 (0.36-1.15)	0.30(0.15-0.58)***	0.31(0.16-0.60)***
	LLIN-only		2.48 (1.67-3.28)	Ref	Ref
	FSH+LLINs	*An. funestus*	0.69 (0.25-1.14)	0.18(0.09-0.36)***	0.20(0.11-0.37)**
	LLIN-only		3.81 (2.29-5.32)	Ref	Ref
	FSH+LLINs	*FedA n. arabiensis*	0.36 (0.12-0.59)	0.22(0.09-0.51)*	0.22(0.10-0.53)***
	LLIN-only		1.67 (1.04-2.30)	Ref	Ref
	FSH+LLINs	*FedA n. funestus*	0.5 (0.15-0.84)	0.16(0.07-0.33)***	0.17(0.08-0.34)***
	LLIN-only		3.22 (1.78-4.66)	Ref	Ref
	FSH+LLINs	*Culicines*	1.14 (0.57-1.71)	0.95(0.48-1.86)ns	0.92(0.46-1.82)*
	LLIN-only		1.24 (0.69-1.81)	Ref	Ref
	Wet	*Anophelines*	6.1 (4.40-7.80)	7.44(3.19-17.38)***	6.12(3.13-11.97)***
	Dry		1.03 (0.53-1.53)	Ref	Ref
	Wet	*An. arabiensis*	2.39 (1.65-3.12)	4.15(2.17-7.93)***	3.39(2.19-7.04)ns
	Dry		0.6 (0.26-0.94)	Ref	Ref
	Wet	*An. funestus*	3.64 (2.28-5.00)	11.56(2.64-50.71)***	9.18(2.93-28.81)**
	Dry		0.42 (0.12-0.72)	Ref	Ref
	Wet	*FedA n. arabiensis*	1.59 (1.01-2.16)	6.48(3.32-12.64)***	5.92(3.28-10.66)***
	Dry		0.26 (0.11-0.40)	Ref	Ref
	Wet	*FedA n. funestus*	3.1 (1.81-4.38)	19.16(3.77-97.23***	14.77(4.19-52.06)
	Dry		0.23 (0.03-0.42)	Ref	Ref
	Wet	*Culicines*	0.88 (0.45-1.31)	0.56(0.30-1.03)ns	0.56(0.30-1.04)ns
	Dry		1.6 (0.86-2.33)	Ref	Ref

***P < 0.001; **P<0.01; * P<0.05, ns = not significant; Ref= reference.

Multivariate analysis after controlling for the seasonality showed 75% [RR=0.25, 95% CI 0.15-0.42, P<0.0001] fewer anopheline mosquitoes in the FSH+LLIN houses (Table 1) compared to control houses. There was a 78% [RR=0.22, 95% CI 0.10-0.53, P<0.0001] reduction in the density of bloodfed *A n. arabiensis.* The number of blood-fed *A n. funestus* was reduced by 83% [RR=0.17, 95% CI 0.08-0.34, P<0.0001] in the FSH+LLIN houses compared to LLIN-only houses.

Seasonal dynamics influenced the mosquito collections. 746 were collected in the rainy seasons and 180 in the drier seasons. Out of the 746, 164 were from the FSH+LLIN houses and 582 from the LLIN-only houses and collections of both anopheline species were reduced significantly. In the dry season, significantly fewer *A n. funestus* were collected in the FSH+LLINs houses, but there was no significant difference in the number of *A n. arabiensis* collected in the seasons; P=0.15 (Table 2). No significant difference was found in the number of culicines collected during the dry and wet seasons [RR=0.56, 95% CI 0.30-1.04, P=0.064].

**Table 2.** Seasonal variation in mean indoor resting densities between FSH+LLINS and LLINs.

		*An. arabiensis*		*An. funestus*	
Season	Treatment	Mean(95% CI)	P-value	Mean(95% CI)	P-value
Dry	LLINs+FSH	0.41 (-0.11-0.92)	0.15	0.13 (0.004-0.25)	<0.0001
	LLIN only	1.33 (0.64-2.03)		0.80 (0.19-1.41)	
Wet	LLINs+FSH	0.75 (-0.05-1.55	0.003	0.25 (-0.55-1.05)	0.0006
	LLINs Only	6.86 (5.24-8.48)		6.40 (3.76-8.48)	

### 3.3 Residual deltamethrin content

The effect of natural UV radiation did not have any effect on the rate of decay of the insecticide on eave nets in relation to the side of the house they were installed. The mean residual deltamethrin content from eave net pieces taken after 12 months was 0.33g/kg and 0.32g/kg for the north and the east side of the house, respectively. There was no significant difference in residual insecticide content for eave net pieces cut from east and north side after 12 months [OR=1.16, 95% CI 0.55-2.45, P=0.70] and 24 months [OR=0.44, 95%, CI 0.16-1.18, P=0.1038]. Further analysis with regard to eave net pieces from all four sides of the house also showed no significant difference in residual deltamethrin content [OR= 0.70 95%,Cl 0.198-2.470, P=0.579]. The two types of door hangers (laminated door streamers and netting material) also showed no significant difference in content after 24 months [OR=1.29, 95%, Cl 0.34-4.84, P=0.71] though laminated door steamers had a 29% higher concentration for the residual deltamethrin content. With regard to door curtains there was no significant difference in residual insecticide content on curtains that were placed in doors facing north or east direction [OR=0.98, CI 0.13-7.25, P=0.71]. Nevertheless, LLIN bednets had nearly the double amount of deltamethrin after 24 months compared to the eave nets (Table 3).

**Table 3.** Deltamethrin residual concentrations in eave net samples or door lamellae after 12 and 24 months of use.

Material	No tested	Months in use	Mean deltamethrin (g/kg)	RR (95% CL)	P-value
Eave north	42	12	0.33(0.25-0.41)	1.16(0.55-2.45)	P=0.70
Eave east	42	12	0.32(0.26-0.38)	Ref	
Eave north	10	24	0.11(0.02-0.20)	0.04(.05-.19)	<0.0001
Eave east	10	24	0.25(0.34-0.46)	0.05(.11-.45)	<0.0001
DC* north	9	24	0.04(0.007-0.07)	0.04(.02-.07)	<0.0001
DC west	5	24	0.06(0.01-0.12)	0.05(.05-.06)	<0.0001
DC south	11	24	0.07(0.03-0.11)	0.07(.03-.15)	<0.0001
Door lamellae	5	24	0.08(0.07-0.23)	0.07(.02-.24)	<0.0001
Bednet	16	24	1.11(1.06-1.16)	1	Ref

* DC = Door curtains.

### 3.4 Residual insecticidal net activity of eave nets

**Figure 5.**
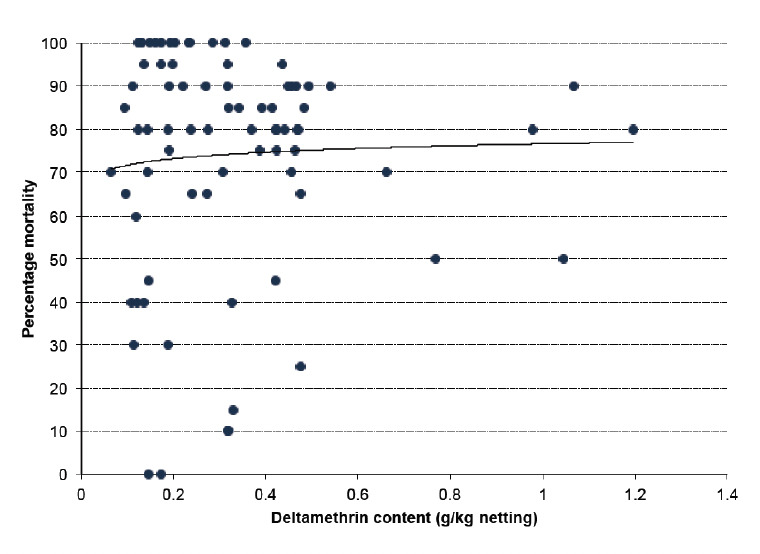
Mortality (% ) from bioassays versus residual deltamethrin content. Line: y = 0.0216 ln(x)+0.766. R^2^= 0.00272.

Pieces of eave nets, window curtains, door nets and laminated door streamers were subjected to bioassays after 12 and 24 months of use in the field. Bioassays with susceptible *A n. gambiae s.s.* Kisumu strain after 12 months on 84 pieces of eave net samples from north and eastern sides of houses (42 pieces from each side) showed a mortality rate of ranging from 50 to 100% after 3 minutes of exposure. When average mortality on sample pieces of nets cut from the north and east side eaves of the houses were compared, there was no significance difference in efficacy [OR=0.81,95%, Cl 0.37-1.73, P=0.58] although pieces from the north side of the house had 19 % lower average mortality compared to samples from the east side. No significant correlation was found between the log residual deltamethrin content and efficacy of the eave net, [OR=1.09, 95%, Cl 0.59-2.02, P=0.079]. Eighty percent (80%) of the eaves nets provided more than 80% mortality after 12 months of use (Figure 5).

Results obtained from the sample nettings collected after 24 months showed that door lamellae provided highest mortality (97 %), followed by bednets (92%). Door nets were the lowest at 76% (Table 4). Door lamellae killed more mosquitoes [OR=11.75(4.37-31.59), P<0.0001] than bednets. The difference observed between eave nets and bednets [OR=1.74(0.68-4.51), P=0.25 and door lamellae and bednets was not statistically significant [OR=0.64, 95% Cl 0.08-5.37, P=0.68].

**Table 4.** Bioassays with susceptible An. gambiae on net samples after 24 months.

Net sample	No. of net sampled	% Knock down after 60 min exposure	% Mortality after 24Hrs
Bednet	13	98.2	92.0
Eave net	24	99.05	95.3
Door curtains	11	60.9	76.0
Door streamers	5	88.52	96.7
Control (untreated net)	1	5.26	0

## 4 Discussion

The use of insecticide-treated curtains to screen the eaves, door(s), and window(s) (here named a fully screened house, FSH) combined with LLINs was compared to houses with LLIN only. FSH+LLINs reduced the number of *An. arabiensis* caught indoors by 85% and *An. funestus* caught indoors by 70% compared to LLINs only. However, the number of culicines found indoors was similar in both arms. Culicines have been reported to enter preferably through windows and doors and not through the eaves which may explain the absence of any difference between the treatments for these species [27].

The combination of FSH+LLINs also provided a high reduction in the number of bloodfed mosquitoes collected indoors, with significant reductions of 84% and 72% for *An. funestus* and *An. arabiensis*, respectively. The difference in control was higher during the rainy seasons than in the dry seasons, when mosquito populations were much smaller and transmission lower even in this area that experiences year-round transmission.

These anopheline species usually find their way into the house through open exits with preference for the eaves [12,13,27,28]. The use of insecticide-treated barriers to screen all entries creates both a physical and a chemical barrier to blood-questing females. The mesh size was too small to allow mosquitoes to penetrate and the insecticide either repelled or killed susceptible mosquitoes that came into contact with it. Our experience from a preliminary test in the same area showed that to avoid mosquitoes from passing a non-treated eave net, the net had to cover every opening thoroughly including the folds of the border of a corrugated roof, which was very time consuming. With a pyrethroid treated net, this was not necessary. Further, untreated nets would have given mosquitoes the possibility to leave the house and bite people elsewhere. The study design did not enable us to assess this effect.

Studies carried out in Mozambique on the use of del-tamethrin-treated shade cloth showed a significant effect on *An. gambiae s.l.,* but there was no significant difference on the population of pyrethroid-resistant *A n. funestus s.l.* [29]. House screening in The Gambia and Ethiopia resulted in reductions of house entry by 43% and 42%, respectively [11,30]. In Ethiopia screening of doors and windows and closing the eaves and walls by mud reduced the overall indoor density of *A n. arabiensis* by 40% [30]. Despite the presence of pyrethroid resistance in the study area the use of FSH+LLINs was able to reduce the indoor resting anopheline population [31].

The quality and precision of the screening process plays an important role in its effectiveness. Lines *et al.* [16] made two field tests, one where nets were fixed carefully and with a high effect, but the time spent on it was evaluated to be too high, and in a second less careful net set-up, its effect on protection was very limited. Therefore, in this study much attention was directed to the preparation of screening material that was easy to set up. The team of 3 trained individuals reduced the set-up time from nearly 1.5 hrs to 30 min. Two persons were fixing the eave nets and one the windows and door nets or laminates. This makes it competitive to IRS in level of time that a team spend per house without having inhabitants to move furniture in and out or cover it with sheets [32,33]. Further, contrary to IRS, it reduces bloodfeeding thus provided personal protection. Finally, other insecticides that can be incorporated into plastic polymer yarns may be used in eave nets, though door hangers and window curtains can only use low-toxicity insecticides like pyrethroids (probably in combination with piperonyl butoxide).

One aspect of preparing the eave net material was adding high levels of UV protection since these materials are more or less exposed to UV. Eave nets are under the roof and therefore, direct exposure to sunlight may only happen at dawn and dusk. Northern eave nets were not directly exposed to the sun. However, after one year no difference was found in residual deltamethrin from eave nets samples of north and east, but losses were significant compared to the indoor used bed nets (Table 4, 24 months). Window curtains and door curtains were fully exposed to sunlight and indeed some of these had very low residual levels of insecticide after 2 years (Table 4). Door laminates, also fully exposed to sunlight, had deltamethrin losses in the same range as the door curtains. Insecticide-treated material degrades more when placed outdoors compared to when used indoors as in LLINs. This suggests a potential barrier for use of deltamethrin-treated materials in the outdoor environment. Since the loss of insecticide was higher with the outdoor nets than with the indoor bednets, the level of UV protection should be upgraded. Alternatively, higher outdoor temperature and air movement through the material may also have contributed to insecticide loss.

Despite these losses, the bioassay results on eave nets showed no correlation between residual insecticidal content and efficacy. It is interesting to note that 12 months after installation mortality was high and even 100% for nets where deltamethrin was low. This suggests that deltamethrin is less soluble in polyethylene matrix and thus migrates to the surface. The lower concentration is in the yarn and is slowly released to the surface. Since it is the surface concentration and not the total insecticide content in the yarn that determines mosquito mortality, this can explain the rather poor correlation between total deltamethrin content and mosquito mortality. After 2 years, eave nets, bednets and door laminates showed increasing effectiveness in that order, but these differences were not significant. Eave nets had an efficacy of 97% after 24 months following 3 minutes exposure tests, which was as good as the bednets. Eave nets do not lose as much deltamethrin as door curtains and can still effectively kill mosquitoes in bioassay. This could be attributed to lack of washing of eave nets, so a large part of the insecticide could still be present at the surface and active. Contrary to that, polyethylene netting material used on the door nets was washed (up to 4 times) and showed a relatively high loss in residual insecticide content. Hence door curtains should be replaced more frequently than eave nets.

This trial confirms that using insecticide treated nets to screen the eaves, windows and doors of houses prevents entry by mosquitoes and can be used to supplement the use LLINs in malaria-endemic areas, especially in areas where the principle vectors bite indoors. Use of insecticide treated materials largely prevents the vector from entering the house while LLINs only protect people already in bed. Eave nets can have other insecticides incorporated than bed nets since they are not exposed to daily contact with people and especially not to babies who may sucking the net, which is a critical toxicological barrier for insecticides applied to bed nets [34]. Unlike bednets, eave nets do not call for daily compliance behaviour and can thus also work in the drier part of the year when bednet use is low, but malaria transmission still on-going. Blocking of eaves might well be one the cheapest of the three options, but schemes for promoting awareness and understanding of these accessible options for household-based control need to be developed and evaluated. The value of this approach is bolstered by the observation that residents of houses with ceilings, screened windows, and especially the combination of both, take advantage of this protection by spending more time indoors at night [28]. These aspects will further be discussed in a separate paper. A larger trial than this should be carried out to show the impact on malaria transmission. A limitation of the study was that no untreated material was used. It was therefore not possible to differentiate between the effect of nets as a physical or a physical plus chemical barrier.

## 5 Conclusions

We have demonstrated that insecticide-treated eave nets, combined with screening of windows and doors significantly reduces house entry by malaria vectors. This approach can be used to supplement the use LLINs in malaria-endemic areas, especially in areas where the principle vectors bite indoors.
